# Recognition algorithm for laboratory protective equipment based on improved YOLOv7

**DOI:** 10.1016/j.heliyon.2024.e36264

**Published:** 2024-08-13

**Authors:** Huijuan Luo, Wenjing Liu, Pinghu Xu, Lijun Zhang, Lin Li

**Affiliations:** a National Center for Materials Service Safety, University of Science and Technology Beijing, Beijing, 100083, China; b Research Institute of Macro-Safety Science, University of Science and Technology Beijing, Beijing, 100083, China; c Department of Innovative Medical Research, Chinese People's Liberation Army General Hospital, Beijing, 100853, China

**Keywords:** Laboratory protective equipment, Target detection, YOLOv7, GAM, NWD metric

## Abstract

In the university laboratory environment, it is not uncommon for individual laboratory personnel to be inadequately aware of laboratory safety standards and to fail to wear protective equipment (helmets, goggles, masks) in accordance with the prescribed norms. Manual inspection is costly and prone to leakage, and there is an urgent need to develop an efficient and intelligent detection technology. Video surveillance of laboratory protective equipment reveals that these items possess the characteristics of small targets. In light of this, a laboratory protective equipment recognition method based on the improved YOLOv7 algorithm is proposed. The Global Attention Mechanism (GAM) is introduced into the Efficient Layer Aggregation Network (ELAN) structure to construct an ELAN-G module that takes both global and local features into account. The Normalized Gaussian Wasserstein Distance (NWD) metric is introduced to replace the Complete Intersection over Union (CIoU), which improves the network's ability to detect small targets of protective equipment under experimental complex scenarios. In order to evaluate the robustness of the studied algorithm and to address the current lack of personal protective Equipment (PPE) datasets, a laboratory protective equipment dataset was constructed based on multidimensionality for the detection experiments of the algorithm. The experimental results demonstrated that the improved model achieved a mAP value of 84.2 %, representing a 2.3 % improvement compared to the original model, a 5 % improvement in the detection rate, and a 2 % improvement in the Micro-F1 score. In comparison to the prevailing algorithms, the accuracy of the studied algorithm has been markedly enhanced. The approach addresses the challenge of the challenging detection of small targets of protective equipment in complex scenarios in laboratories, and plays a pivotal role in perfecting laboratory safety management system.

## 1 Introduction

With the advent of the post-epidemic era, the laboratories of scientific research institutes as important locations for scientific research and learning, have been reopened. In large research institutes, laboratory personnel should wear personal protective equipment (PPE) prior to conducting experiments. However, due to high personnel turnover rates in laboratories, some laboratory personnel have insufficient safety awareness resulting from inadequate safety training. They ignore laboratory safety rules, and enter the laboratory without wearing personal protective equipment. This action posed significant risk to the safety of laboratory personnel [1].

Traditional laboratory safety research mainly focuses on updating instruments and equipment, improving harmful environment, strict laboratory access, and improving laboratory safety management system [2]. However, studies on violations caused by people's weak safety awareness and non-compliance with laboratory safety rules are few [3,4]. Standardized wearing of personal protective equipment is important to protect laboratory personnel from harmful environment or accident injuries during the experiment, which is often ignored by laboratory personnel in actual operation [5]. What's more, with the complexity of laboratory equipment layout and personnel flow, it is difficult to use traditional monitoring methods to achieve the management of individual protective equipment in complex laboratory situations. Therefore, an effective monitoring and testing mechanism is particularly important to protect laboratory personnel and improve the level of laboratory safety management.

To standardize the management of personal protective equipment wearing in complex laboratory conditions and avoid casualties caused by workplace accidents, research work on intelligent detection for PPE has been conducted both domestically and internationally. Santiago Barro-Torres et al. [6] used body area network sensor technology to detect PPE near certain parts of the body, so as to monitor the wearing of PPE of construction workers in real time. Based on computer vision, Venkata Santosh Kumar Delhi et al. [7] applied transfer learning to the single-stage object detection algorithm YOLOv3 [8] to detect the wearing compliance of construction workers' hard hats and protective jackets. Ju-Yeon Lee et al. [9] designed a workplace safety helmet classification model based on deep learning method to detect the standard wearing of construction workers' safety helmets. The model used existing helmet training data in two steps and had higher recognition accuracy compared to using only one model. Faming Gong et al. [10] used transfer learning to locate the target area based on the spatial relationship of the key points of the human body, so as to realize the detection and recognition of hard hats and overalls during Marine operations. Nipun D. Nath et al. [11] proposed three deep learning models based on YOLOv3 to verify the standard wearing of safety helmets and vests by construction workers respectively. The models showed better performance when a single convolutional neural network framework was used to detect a single worker. Jiaqi Li et al. [12] created a YOLOv5-based model for the detection of helmets and safety harness's hook. Without affecting the normal behavior of individuals, the model realizes the discrimination of PPE misuse, which improves the efficiency of safety management to a certain extent.

Although progress has been made in detecting PPE, there are still deficiencies in safety protection application.

(1) As hardware integrated into workwear, the sensor device focuses on "wear" rather than "standardized wear" when detecting PPE. It is not possible to detect workers' compliance with the wearing of PPE, and its widespread use is not conducive to cost savings.
(2) With the continuous updating of computer vision algorithms, the current means of target detection applied to PPE focuses on large targets, while the detection of PPE for small targets is rarely involved, and there is relatively little research on the standardization of wear. The model still has room for improvement in recognition accuracy and detection rate.

In order to be more suitable for the practical application of PPE testing in the laboratory, this paper takes National Center for Material Service Safety (NCMS) of University of Science and Technology Beijing (USTB) as an example. When conducting experiments in the laboratory, it is essential to wear laboratory protective equipment such as safety helmets, goggles, and masks.

In this paper, based on the phenomenon of non-standard wearing of laboratory protective equipment, for the problem of difficult detection of small targets in laboratory protective equipment, we propose a detection method based on the improvement of YOLOv7 [13], which is applicable to the detection of small targets in laboratory protective equipment. And construct a semi-self-built laboratory protective equipment dataset, the final detection results are comprehensively compared with other target detection algorithms, reducing the training loss of the model while improving the detection accuracy of the model. This method effectively solves the problem of difficult detection of small targets of protective equipment in complex laboratory scenarios, and provides a more intelligent and innovative solution to improve the level of laboratory safety management. This study contributes to the further improvement of the laboratory safety management system.

The principal contributions of this paper are as follows.

(1) A laboratory protective equipment dataset is created. The dataset encompasses a variety of attributes, including pixel occlusion, different light illuminations, flipping, and conventional bounding box labeling. This solves the current lack of PPE small target datasets, provides rich and high-quality data support for in-depth research of existing methods, and promotes applied research on PPE small target detection in multiple fields and from multiple perspectives.
(2) The laboratory protective equipment small target detection algorithm was designed. Aiming at the problem of difficult detection of small targets, based on YOLOv7, several algorithms are optimized and combined to design an algorithm for detection of small targets in laboratory protective equipment. The sophistication and effectiveness of the algorithm are verified through experiments, and more accurate detection and identification of laboratory protective equipment is realized.
(3) Contributes to the enhancement of laboratory safety management systems. The objective of this paper is to present an intelligent, real-time detection system for laboratory protective equipment. This system is designed to provide more intelligent and innovative solutions to improve the level of laboratory safety management. This study contributes to the further improvement of the laboratory safety management system.

The organization of the paper is as follows. The second section briefly introduces common object detection algorithms in deep learning, including the structure of YOLOv7 used in this paper. The third section introduces the proposed laboratory protective equipment identification algorithm based on the improved YOLOv7, including detailed improvement strategies. The fourth section is the establishment of laboratory protective equipment data set, including data set composition and its preprocessing. The fifth section analyzes the model loss and test accuracy for target detection before and after algorithm improvement. Additionally, the efficiency of the algorithm in this paper is verified through comparison and ablation experiments. The last section puts forward the discussion, including implication, limitations and future work, of this paper.

## 2 Commonly used target detection algorithms

### 2.1 Commonly used algorithms

Deep learning based target detection algorithms fall into two main categories. One class is the Region of Interest (ROI) based target detection algorithm, called Two-stage algorithm. The Two-stage algorithms have been developed after Region Convolutional Neural Network (R–CNN), Spatial Pyramid Pooling in Deep Convolutional Networks (SPP-Net) [14], Fast R–CNN [15], Faster R–CNN [16], and Mask R–CNN [17], their training and testing time are decreasing and training accuracy is improving. However, the algorithm itself contains multiple cut steps, which will take up a large storage and computation overhead during operation, and its time complexity is not as good as that of single-stage algorithms. And due to the large size of the model, the performance requirements of the camera are extremely high, which makes it difficult to realize universal application and promotion. The other category is One-stage algorithm. The most representative algorithm is the YOLO, which is an end-to-end target detection algorithm based on a regression strategy. The YOLO simplifies the target detection problem into a regression problem. Since the YOLO does not have a region recommendation stage, it has a higher detection speed compared to the two-stage algorithm [18].

With the continuous development of computer vision technology, YOLO is widely used in various fields with its better real-time performance. In medical identification, Xinbei Jiang et al. [19] proposed a medical mask detection method based on improved YOLOv3, which improves the detection accuracy and model robustness of medical masks. In the field of elderly fall recognition, Jun Peng et al. [20] improved the backbone network and PANet of YOLOv5, which enhanced the localization ability of the elderly fall detection model and reduced the detection false alarm rate. In the field of classroom behavior recognition, Wentian Niu et al. [21] proposed an enhanced skeletal recognition system based on YOLOv5 for human skeletal movement recognition in order to accurately detect behaviors such as raising hands, standing, and dozing. Yan Zhou [22] proposes a novel object detection model based on YOLOv5 for real-time mask wearing detection. In the field of intelligent transportation, Avinash Padmakar Rangari et al. [23] designed a YOLOv7-based intelligent control model for traffic signals, which achieves to some extent the purpose of allocating time and managing traffic signals in complex traffic scenarios.

Deep neural networks have shown excellent detection performance for large targets in various fields, but they are still lacking in small object detection (SOD). Small targets have always been the difficulty in the field of target detection due to the characteristics of few pixels, aggregation, and weak feature representation. Faced with this status quo, Gong Cheng et al. [24] starting from the small object dataset, believed that one of the reasons for the slow breakthrough progress in the field of SOD detection is the lack of a benchmark dataset for small objects, and then independently constructed a large-scale SOD dataset for the transportation field (SODA), and evaluated its performance by using mainstream target detection methods (FCOS, Faster R–CNN, etc.), but failed to improve the problem of low SOD accuracy at all. Jie Zhang et al. [25] detected small targets in remote sensing and transportation domains by the improved YOLOv4, and tested it on various datasets, which basically achieved more than 70 % accuracy. Xiang Yuan et al. [26] proposed the CFINet two-stage small target detection framework based on Faster R–CNN, which improved by 1.8 % compared with the baseline model on SODA dataset. Although researchers have made some progress in small target detection, there are still some shortcomings.

(1) Dataset limitations. Existing SODA datasets are small and focus more on transportation, which is not enough to comprehensively evaluate and improve the performance of small target detection algorithms.
(2) Limited algorithm performance improvement. More effective algorithms are needed to solve the problem of small target detection.
(3) Practical application challenges. Small targets are usually affected by factors such as occlusion and light changes, which can reduce the accuracy of detection.

### 2.2 YOLOv7 model

YOLO is used in various fields nowadays, which has high research significance and industrial application value. Among them, YOLOv7 can fuse the features of different layers due to its complex network structure layers, which is favorable for small target detection. Therefore, YOLOv7 is chosen in this paper for small target detection of laboratory protective equipment.

**Fig. 1:**
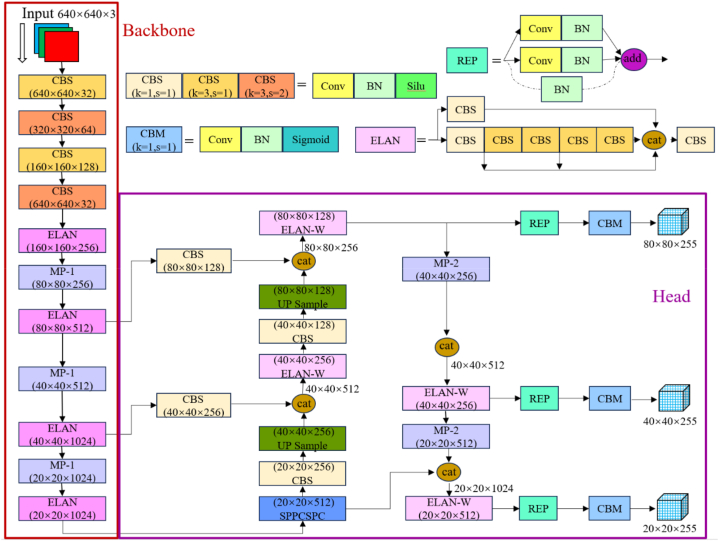
Network structure of YOLOv7.

As shown in Fig. 1, the YOLOv7 network consists of Input, Backbone, and Head, three-part structure. When target recognition is performed, the input side preprocesses the input picture. Backbone is a classification network, which mainly extracts information features from the input picture, and the obtained feature map is passed to the Head network. As the classifier and regressor of YOLOv7, Head network fuses the shallow graphical features with the same number of channels and size of Backbone by adjusting the channel and feature scale to avoid important information loss to some extent. Finally Head network predicts the class and location of the target after reciprocal feature extraction, channel transformation and fusion.

## 3 Improvements to laboratory protective equipment detection model

YOLOv7 can perform multi-scale target prediction and fuse features from different layers, thus mitigating the loss of small-target features brought about by the layer-by-layer increasing receptive field and promoting the improvement of small-target detection accuracy. Safety helmets, goggles, and masks are all small targets, these sample targets are often only a few pixels in size in the images intercepted under surveillance cameras, and the recognition accuracy of existing target detectors on such targets needs to be improved. This paper introduces the attention mechanism, as well as the optimization of the loss function, based on the YOLOv7 network structure to improve the feature extraction ability of YOLOv7 on small targets.

### 3.1 GAM

The attention mechanism in neural networks is a resource allocation scheme that allocates computational resources to more important tasks while solving the problem of information overload in the case of limited computational capacity. In recent years, the attention mechanism has been widely used in different types of deep learning tasks such as image processing, speech recognition, natural language processing, etc. In the process of processing a large amount of input information in the neural network model, the use of the attention mechanism can be done to improve the efficiency of the neural network and obtain a relatively high accuracy by selecting only some key input information for processing. In principle, the attention mechanism model is divided into channel attention model, spatial attention model and hybrid attention model. In general, channel-attentive and spatial-attentive mechanisms have been shown to improve the accuracy of the original model more significantly in target detection. However, these mechanisms utilize visual representations from a limited number of receptive domains, which accordingly leads to information reduction and dimensional separation, ignoring the importance of preserving channel and spatial aspects to enhance cross-dimensional interactions. Typical representatives of hybrid attention mechanisms: the Convolutional Block Attention Module (CBAM) [27] and the Global Attention Mechanism (GAM) [28], similar to the GAM, the CBAM, by considering the feature information in the spatial and channel dimensions, adaptively selects and weights different parts of the feature map, so as to effectively extract relevant features in space and channel and improve the network's ability to focus on the target region. However, laboratory protection equipment under video surveillance has the characteristics of aggregation of multiple categories of small targets and weak feature representation, etc., CBAM is difficult to model the global contextual information and better capture the relationship between small targets and the environment of the laboratory.

Therefore, GAM, as a hybrid attention model, combines the advantages of the first two attention models to improve the performance of deep neural networks by reducing information dispersion and strengthening the global interaction representation, which is suitable for the detection of laboratory protective equipment.

Due to the large area of the laboratory, the number of large equipment, the detection object of the surveillance video exists far from small targets and is relatively fuzzy, which affects the feature extraction of the YOLOv7 model for the laboratory protective equipment, resulting in a low accuracy rate of laboratory protective equipment recognition. In this paper, the GAM is integrated into the YOLOv7 network to extract local features while taking global features into account, in order to improve the model's feature extraction ability for targets in complex scenes.

**Fig. 2:**
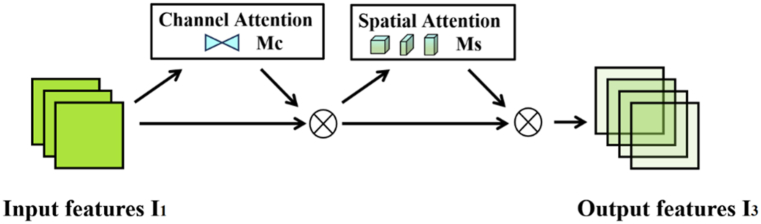
Processing flow of GAM.

GAM builds on CBAM by redesigning the sub-modules. The attention operation can capture important features from the channel, spatial height, and spatial width three dimensions at a time, it can better amplify the interaction of dimensions to avoid losing some Cross-dimensional information. The whole working principle of GAM is shown in Fig. 2. In the processing flow, it mainly includes three parts: input feature mapping, intermediate state and output, in which the intermediate state mainly includes the channel attention sub-module and the spatial attention sub-module.

**Fig. 3:**
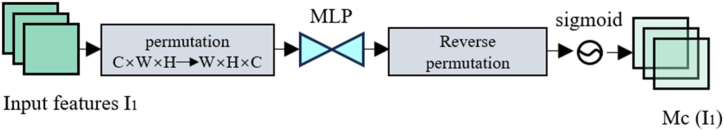
Structure of channel attention sub-module.

The channel attention sub-module is shown in Fig. 3. In the channel attention two layers of MLP perceptron are used to enhance the feature information interaction between channels and to prevent gradient vanishing and gradient explosion between layers using ReLU activation function. In the first layer of MLP perceptron, the number of channels is compressed to reduce the computation in the attention mechanism, followed by adjusting back to the original number of channels in the second layer of MLP perceptron and generating the channel weight coefficients using the sigmoid function, which are multiplied with the input feature maps in a weighted manner. The specific calculation process is as in Equations (1), (2), (3).

(1) $$I_{1}\in R^{C\times H\times W}\text{,}$$

(2) $$y=w_{1}I_{1}^{T}+b_{1}\text{,}$$

(3) $$I_{2}=M_{C}\left(I_{1}\right)⊗I_{1}=\text{sigmoid}\cdot {\left[I_{1}\cdot \text{ReLU}\left(w_{2}y+b_{2}\right)\right]}^{T}\text{,}$$

where I_1_ is the input feature map, I_2_ is the channel attention output feature map, C, H, and W indicate the number of channels, picture height, and picture width, respectively, w_1_, w_2_ and b_1_, b_2_ are the random initial weight values and bias terms of the two-layer MLP perceptron, respectively. M_C_ is the channel attention map, and ⊗ denotes multiplication by elements.

**Fig. 4:**
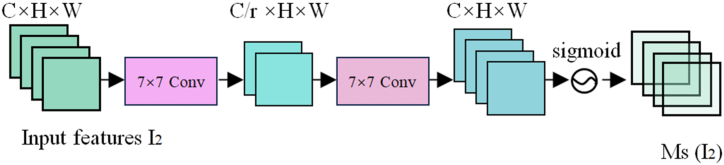
Structure of the spatial attention sub-module.

Although channel attention has been implemented to attend to different feature maps in the channel dimension, it is not able to attend to the local information of feature maps in the spatial dimension. The spatial attention mechanism utilizes the spatial relationship between features to generate spatial attention maps to focus on the more important regions of the context. Its structure is shown in Fig. 4.

To focus the spatial information, two convolutional layers are used for spatial information fusion. Neither of the two convolutional layers performs down sampling operation, which reduces the loss of spatial information. Firstly, the feature map is compressed by 7 × 7 convolution to fuse the spatial information of the feature information at the same position in different channels to reduce the computational overhead. Then after the second layer of convolution, the number of channels is increased to keep the same as the original number of channels. Finally, the sigmoid function is used to generate spatial weight coefficients, which are multiplied with the input feature map to do the weighting process, to realize the allocation of attention to different regions of the feature map. The specific process is calculated as Equation (4):

(4) $$I_{3}=M_{S}\left(I_{2}\right)⊗I_{2}=\text{sigmoid}\left[\text{Conv}\left(\text{BN}\;\text{ReLU}\left(I_{2}\right)\right)\right]\text{,}$$

where I_3_ is the global attention output feature map, Conv for convolution and BN for batch normalization, and MS is the spatial attention map.

### 3.2 NWD

The total loss function of YOLOv7 algorithm consists of box position loss, confidence loss and classification loss together, and the total loss function formula is as Equation (5):

(5) loss=box_loss+cls_loss+obj_loss.

Where box_loss is the bounding box loss, which is used to measure the difference between the predicted box position and the true box position in the target detection model. When box_loss is low, it indicates that the model is able to accurately predict the location of the target. Obj_loss is the target confidence loss, which is used to measure the accuracy of the target detection model in determining whether a target is present in the image. When obj_loss is low, it indicates that the model is able to correctly identify the target in the image. Cls_loss is the classification loss, which is used to measure how accurately the target detection model categorizes different categories. When cls_loss is low, it indicates that the model has high accuracy in distinguishing between different categories.

Aiming at the problem that the traditional IoU metric calculation is sensitive to small target position deviation, this paper proposes to introduce Normalized Gaussian Wasserstein Distance (NWD) [29] in the regression loss function to measure the similarity between the predicted bounding box and the real target bounding box. The IoU metric of traditional target detectors calculates the degree of overlap between the calibrated frame and the predicted frame. Goggles and masks have fewer pixels and are discrete with respect to changes in scale, and small positional deviations can lead to a significant decrease in the value of IoU, but the use of NWD mitigates this sensitivity. In this paper, in order to better describe the weights of different pixels in the bounding box, the bounding box is modeled as a two-dimensional Gaussian distribution, and NWD is used to measure the similarity of the derived Gaussian distribution. For the horizontal bounding box R= (c_x_, c_y_, w, h), where (c_x_, c_y_) are the centroid coordinates and w and h are the width and height respectively model R as a Gaussian distribution N(μ,Σ), where μ and Σ are described in Equation (6):

(6) $$\mu =\left[\begin{array}{c} C_{X}\\ C_{Y} \end{array}\right],\Sigma =\left[\begin{array}{cc} \frac{w^{2}}{4} & 0\\ 0 & \frac{h^{2}}{4} \end{array}\right]\text{.}$$

Then the Wasserstein distance is used to compute two Gaussian distribution distances between the bounding box R_1_ = (c_x1_, c_y1_, w_1_, h_1_) and the bounding box R_2_ = (c_x2_, c_y2_, w_2_, h_2_). Then the square of the Wasserstein distance between two Gaussian distributions N1 and N2 is as Equation (7):

(7) $$w_{2}^{2}\left(N_{1},N_{2}\right)=\left\|\left({\left[c_{x1},c_{y1},\frac{w_{1}}{2},\frac{h_{1}}{2}\right]}^{T},{\left[c_{x2},c_{y2},\frac{w_{2}}{2},\frac{h_{2}}{2}\right]}^{T}\right)\right\|2^{2}\text{.}$$

where N_1_, N_2_ are Gaussian distributions based on R_1_, R_2_ respectively.

Since this distance metric does not directly measure the similarity between the bounding boxes R_1_ and R_2_, it is subjected to an exponential form normalization operation to obtain a new metric NWD as Equation (8):

(8) $$\text{NWD}\left(N_{1},N_{2}\right)=\mathrm{EXP}\left(‐\frac{\sqrt{w_{2}^{2}\left(N_{1},N_{2}\right)}}{C}\right)$$

The C in Equation (8) is a constant closely related to the dataset. Compared with the IoU metric, NWD can respond more smoothly to changes in target location. Regardless of whether the targets overlap or not, the distribution similarity can be measured; moreover, NWD is less sensitive to targets of different sizes and is more suitable for measuring small targets.

**Fig. 5:**
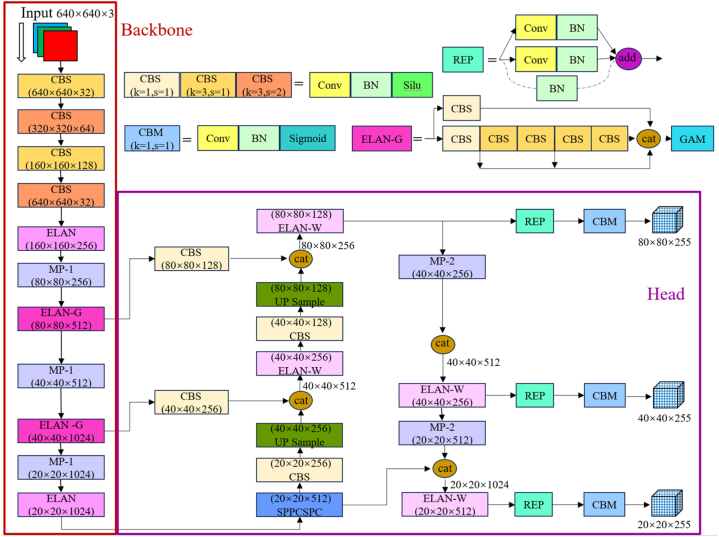
Improved YOLOv7 network structure diagram.

In the improved algorithm, GAM is added to the ELAN module of the YOLOv7 backbone network to construct a kind of ELAN-G module that simultaneously takes into account both global and local features. This enables the network to effectively capture local features while taking into account global features, and improves the network's ability to extract comprehensive target feature information in experimental scenarios. The NWD small target metric is introduced to replace the CIoU loss function of the probe head to improve the model's ability to recognize small targets of the experimental protective equipment. The improved YOLOv7 network structure is shown in Fig. 5.

## 4 Analysis of laboratory scenarios

### 4.1 Creation of datasets

NCMS consists of multiple experimental station, and in this case we have chosen the multiphase flow corrosion experimental station as the site for gathering of this dataset. The multiphase flow corrosion experimental station is a flexible, single-story building containing large, multi-functional test loop pipes specifically designed to simulate and detect the flow pattern and corrosion process of multiphase flow. To ensure that the dataset reflects real and complex laboratory scenes as accurately as possible and prevent overfitting in test results, the dataset comprises three methods: video camera recording, interception by the laboratory's surveillance camera, and recalibration of specific section of the online public dataset. The surveillance camera had 1080P pixels and used Canon EOS M50 Mark2 II with the pixels set to S2 3.8M 1600P. The purpose of the test is to determine the standardization of helmets, masks, and goggles for personnel entering the designated area. The human head, helmet, mask, and goggles were labeled using LabelImg. The initial labeled images numbered 1200, and through data preprocessing, we obtained semi-autonomous datasets that were uniformly distributed. The Training set include2031 images, the validation set consists of 431 images, and the testing set has 293 images. The distribution is set at a ratio of roughly 7:2:1.

The Laboratory Protective Equipment Dataset is a dataset designed to accurately capture personal protective equipment in complex laboratory scenarios. Laboratory protective equipment has significant variability in terms of category, scale, occlusion, and appearance. The dataset contains 4 detection categories, 2755 images, and a total of 33,895 instances in the ,450 human heads, 10,579 helmets, 4515 pairs of goggles, and 4351 masks). The number of experimenters contained in a single image: as few as 1 and as many as 12.

The target instances of the dataset are divided into three classes according to their absolute scaling: small about 75 % (pixel area within 1024), medium about 20 % (1024–9126 pixel area), and large about 5 % (9126–40,000 pixel area). In addition to the regular bounding box labels, targets are provided that include attributes such as pixel occlusion, varying illumination, and flipping. This provides rich and high quality data support for a thorough investigation of existing methods.

### 4.2 Data preprocessing

**Fig. 6:**
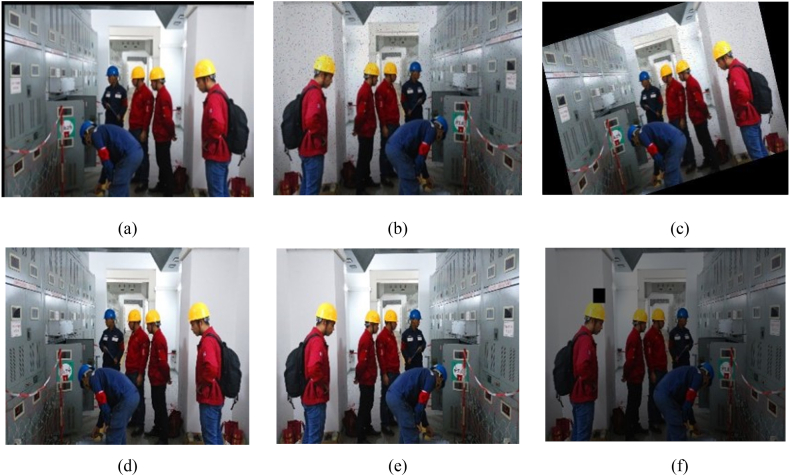
Visualization of image enhancements: (a) Original; (b) Mirror; (c) Color transform and erase on the fly; (d) Translation and blur; (e) Mirror and noise; (f) blur, noise and rotation.

In deep learning tasks, better detection is achieved with larger and high-quality data. Within this laboratory, there is a large site area with various facilities and equipment. Certain large equipment requires movement for operation, and some areas have insufficient lighting. To address complex background interference with detection targets, lower network overfitting probability, and improve model learning ability, preprocessing the laboratory dataset is necessary. In this paper, we use the PyTorch framework and related OpenCV libraries to expand the laboratory dataset through a data expansion strategy [30]. We also implement the random erasure strategy [31,32] to provide a more complex data characterization. Data augmentation is primarily conducted on the training, validation, and test sets using techniques such as flipping, angle rotation, noise transformation, color transformation, and pixel replacement. This leads to enhanced model generalization ability. At the same time, the data enhancement improves the match between the training model and the clarity of the traditional laboratory surveillance video, while also reducing excessive resource consumption during the subsequent real-time processing. Fig. 6 illustrates examples of the effects of data enhancement.

## 5 Experimentation and analysis

### 5.1 Experimental preparation

The experiment utilizes a workstation running on the Windows 10 operation system, powered by the PyTorch deep learning framework and GPU acceleration tool CUDA11.1. Refer to Table 1 for further details on the specific training environment configuration.

**Table 1:** Hardware configuration and model parameters associated with the experiment.

Name	Configuration/Parameters
GPU	NVIDIA RTX A6000
CPU	Core i7-8280
Memory	256 GB
System Environment	Python 3.8
Optimizer	SGD
Learning Rate	0.01

To better align the training model with the practical use of a 1080P surveillance camera, the model's image input size is now set to 960 × 960, with a batch size of 32 and a total of 200 training rounds.

### 5.2 Evaluation indicators

This paper conducts multi-category small target detection to assess the detection efficacy of YOLOv7 for laboratory helmets, masks, and goggles. The paper uses detection accuracy and rate, measured through Precision, Recall, Mean Average Precision (mAP), Micro-F1 Score, and Frames Per Second (FPS). As the model's evaluation index. The mAP assesses whether laboratory protective equipment is worn effectively, while FPS gauges the model's test inference speed.

In Equations (9–13), TP represents the count of correctly recognized detection frames, FP represents the count of incorrectly recognized detection frames, FN represents the count of undetected correct targets, c represents the count of detected categories, AP represents the average value of single-category accuracy, mAP represents the average value of the total average accuracy of the categories, reflecting the model's average of the effectiveness of the recognition of all the categories, and Micro-F1 represents the reconciled average of Precision mean and Recall mean under multi-category target detection, which weighs the Precision and Recall metrics in order to assess the stability of the model, where, n denotes the number of images processed by the model, and t stands for the time consumed.

### 5.3 Results of the experiments

#### 5.3.1 Analysis of model losses

**Fig. 7:**
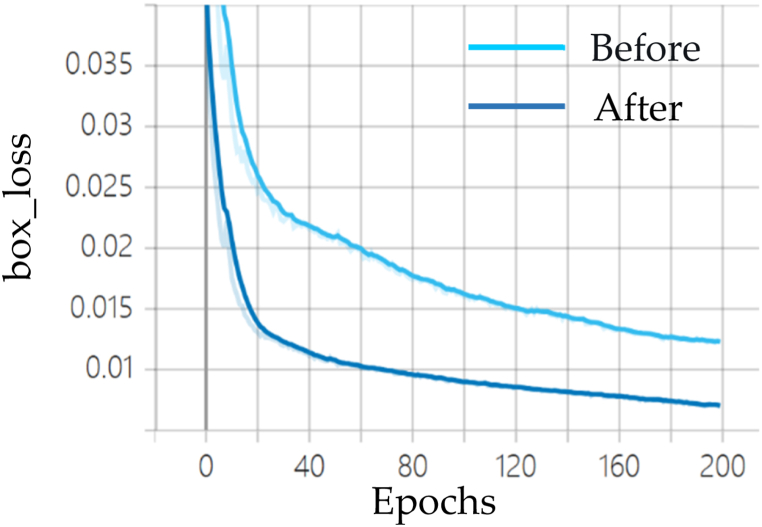
Comparison of bounding box loss before and after YOLOv7 improvement.

**Fig. 8:**
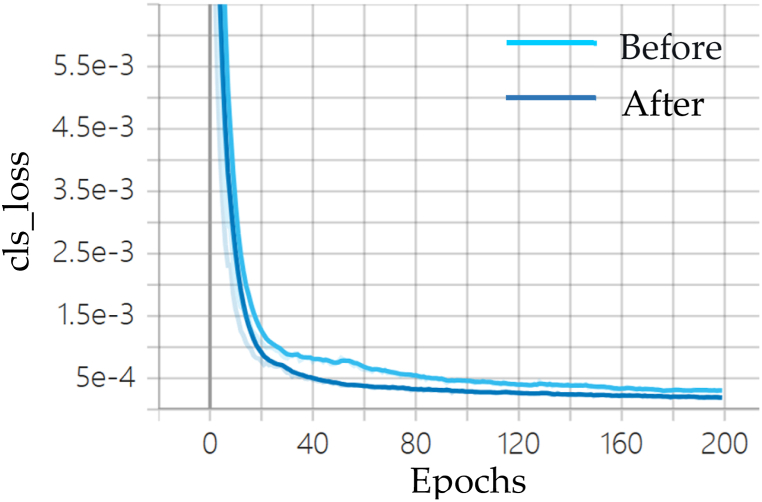
Comparison of classification loss before and after YOLOv7 improvement.

**Fig. 9:**
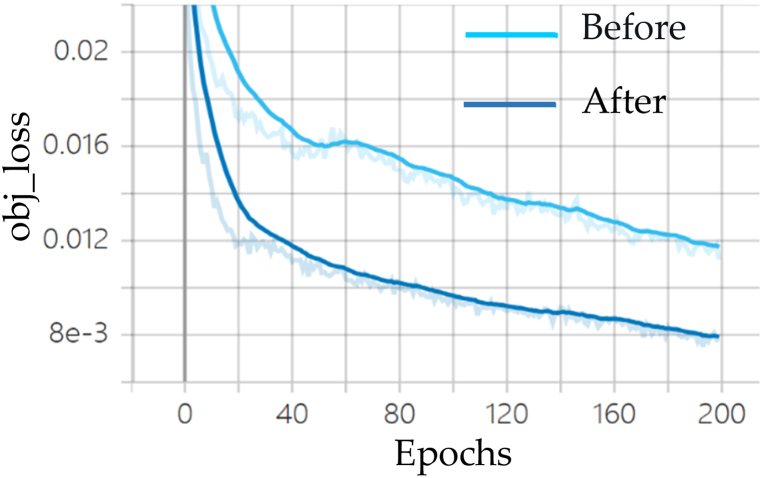
Comparison of confidence loss before and after YOLOv7 improvement.

As shown in Fig. 7, Fig. 8, Fig. 9, the localization loss, classification loss, and confidence loss of the YOLOv7 before and after the improvement under the laboratory protective equipment dataset, respectively. With the rise in the number of iterations, the three loss curves will eventually reach the convergence state. But the improved loss value is smaller compared to the pre-improvement. The overall stability has been improved. So it can be directly concluded that compared with the original algorithm, the loss curves of the improved algorithm can achieve faster convergence and smaller loss values. The use of NMD to optimize the loss function metric has more important significance for the performance improvement of the network model.

#### 5.3.2 Analysis of test accuracy

**Fig. 10:**
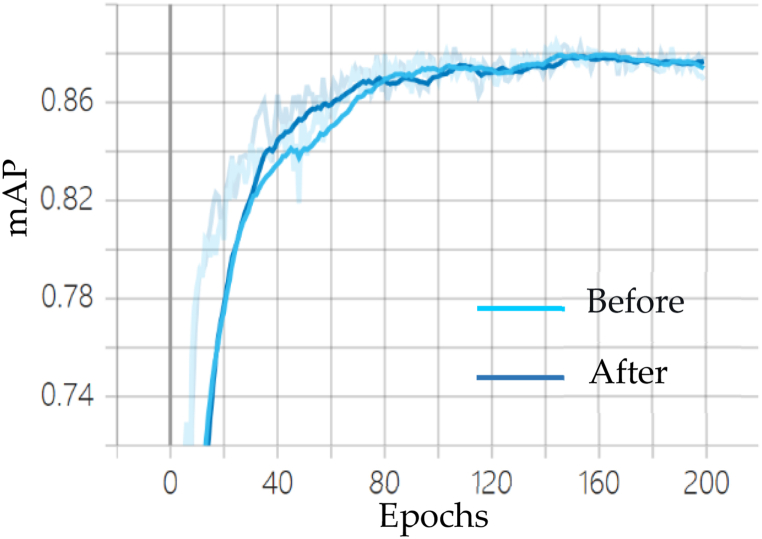
Comparison of mAP before and after YOLOv7 improvement.

**Fig. 11:**
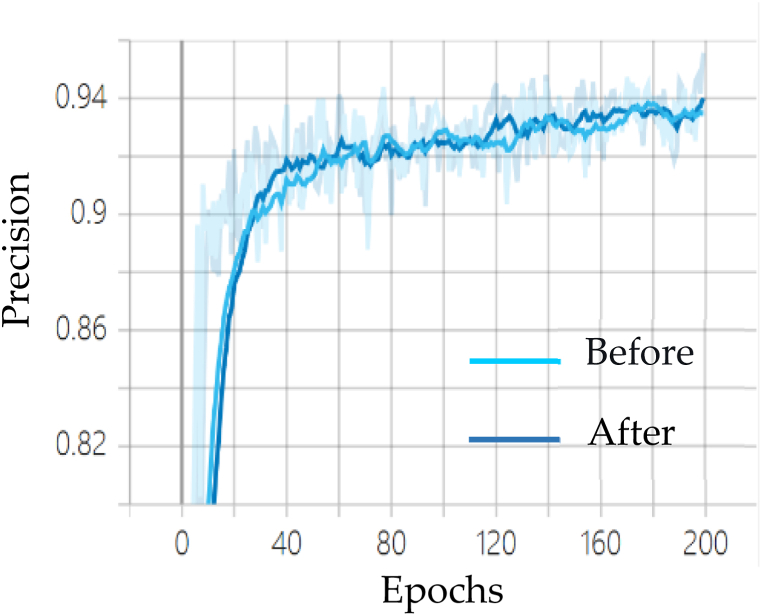
Comparison of Precision before and after YOLOv7 improvement.

**Fig. 12:**
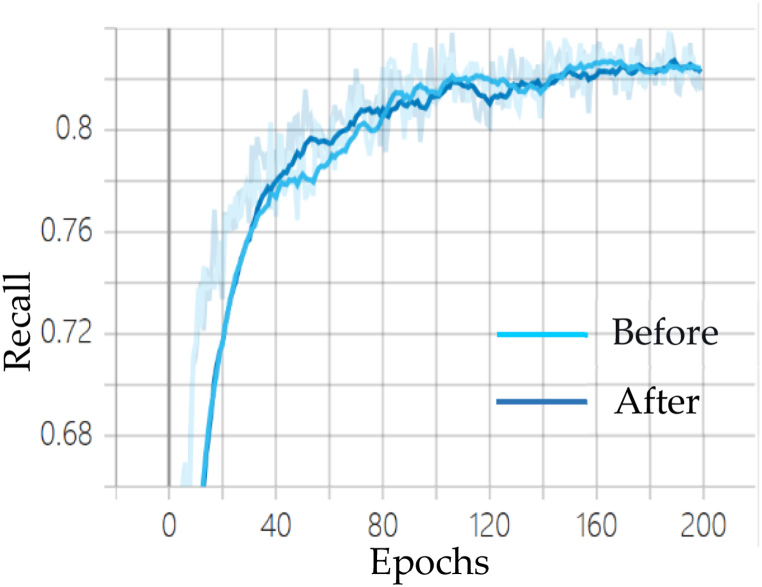
Comparison of Recall before and after improvement of YOLOv7.

As shown in Fig. 10, Fig. 11, Fig. 12, the comparison curve plots of the mean Average Precision (mAP), Precision (P), and Recall (R) of the YOLOv7 algorithm before and after the improvement under the laboratory protective equipment dataset are shown, respectively. It can be seen that compared with the original model, the mAP curve of the improved algorithm reflects faster convergence compared to the original model, and the convergence ability on the P and R curves is also slightly stronger than the original model.

**Fig. 13:**
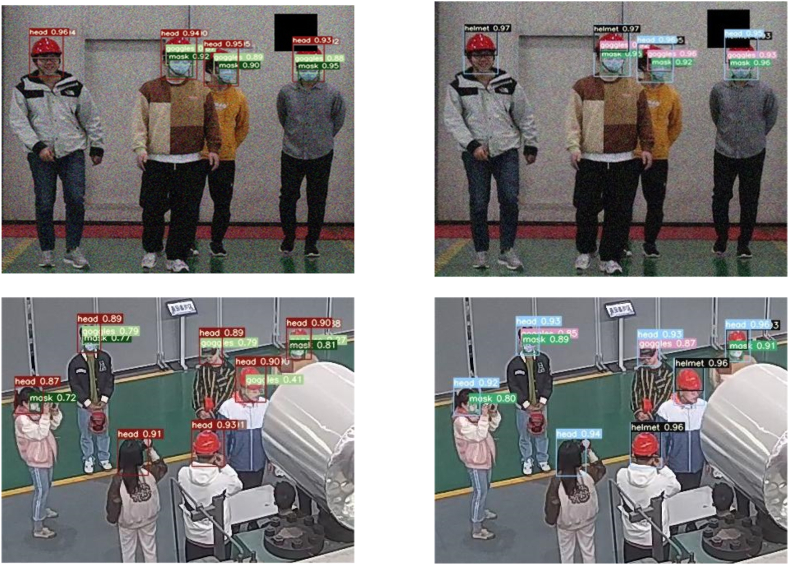
Comparison of accuracy visualization before and after YOLOv7 improvement.

To further prove this paper's algorithm reliability and provide a clearer comparison of before and after improvement of the algorithm, this paper randomly extracts images from the video frames of the surveillance camera to perform real-time prediction inference in the before and after improved models respectively. After the inference, the redundant background of the image is truncated and only the target region is retained, so as to compare the predicted probability values more intuitively and clearly. As shown in Fig. 13, the left figure is the prediction accuracy effect of the original model of YOLOv7, and the right figure is the prediction inference effect of the improved algorithm in this paper. Comparison of the visualization effect can be concluded that this paper's algorithm reflects better prediction accuracy in real-time detection of small targets of laboratory protective equipment, and has higher stability and reliability than the original algorithm.

#### 5.3.3 Analysis of wear regularity detection

**Fig. 14:**
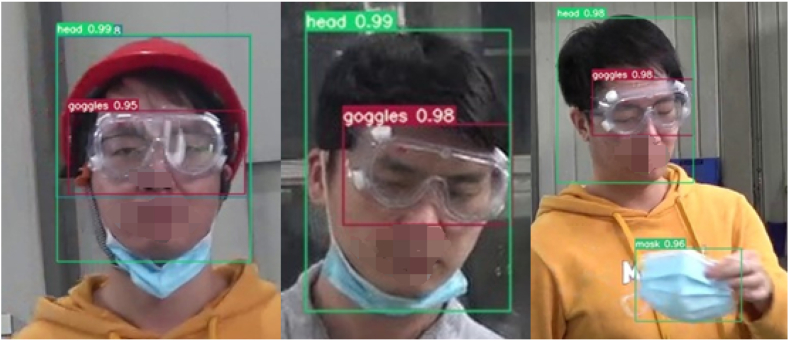
Irregular wearing of masks.

**Fig. 15:**
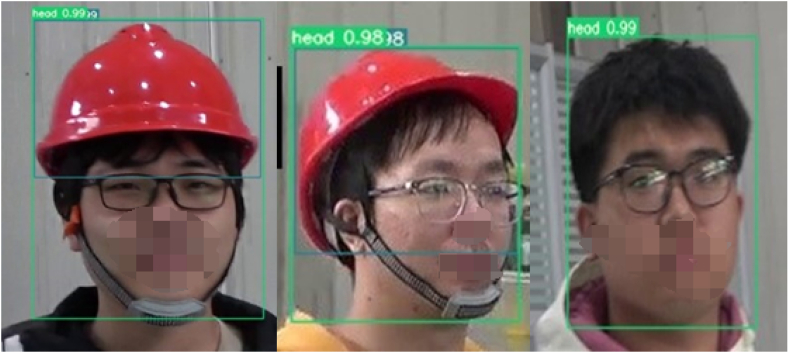
Wearing glasses without goggles.

**Fig. 16:**
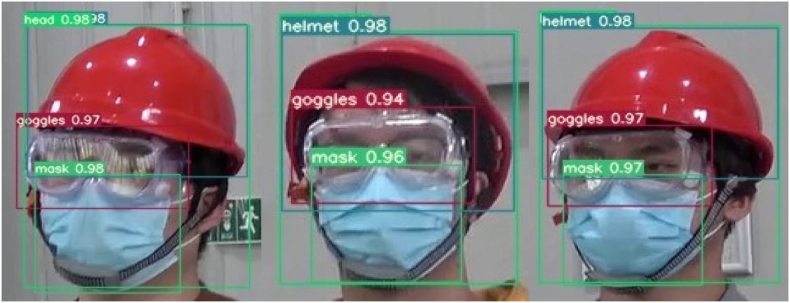
Laboratory protective equipment wear specifications.

In section 4.1, we have already mentioned that the detection purpose of the laboratory protective equipment detection model is whether the laboratory personnel are standardized to wear helmets, goggles and masks. Since eyeglasses and goggles are the same small target, they have some similarity and are easy to be confused during detection. For this reason, in the data processing stage we have collected some video images of laboratory personnel wearing glasses instead of goggles as a control group and become a control dataset for training, validation, and testing in order to reduce the false alarm rate when testing the reasoning of the laboratory protective equipment model. In terms of standardized wear, we stipulate that helmets, goggles, and masks are all standardized when they are worn on the human head, i.e., when the detection frame of the protective equipment is inside the detection frame of the human head, and the rest are "not standardized". Regarding the wear compliance of the laboratory protective equipment, Fig. 14, Fig. 15, Fig. 16 show the visualization of the inference results of the model test taken from the surveillance video. The inferred images truncate the redundant background and retain only the target area for a more intuitive comparison of the predicted probability values. By comparing the three images, we can visualize that the model reflects better accuracy and lower false alarm rate when detecting not standardized in the wearing of laboratory protective equipment.

In this paper, the improved YOLOv7 model is implemented without the help of additional machine learning network frameworks, considering the time and space complexity of the model. In terms of accuracy and false alarm rate, the model achieves precise detection of laboratory protective equipment while saving computational resources and security management costs.

Through the analysis of the experimental results we conclude that the addition of GAM global attention mechanism to the YOLOv7 backbone network, coupled with the optimization of the loss function by NWD, which takes into account both global and local features, effectively improves the feature extraction capability of the network for small targets, and combines the learning strategy of SGD optimizer and cosine annealing. Eventually, compared with the original model, the improved model has smaller training loss, faster convergence effect, higher recognition accuracy, and reflects better stability and generalization ability when performing wearability specification detection of laboratory protective equipment. This reflects the advanced nature of the present algorithm.

### 5.4 Comparative experiments

In order to further prove the advancement of this paper's algorithm for laboratory protective equipment detection, this paper compares it with the current mainstream target detection algorithms RetinaNet [33], Faster-RCNN, FCOS [34], YOLOv5, YOLOv7, and YOLOv7-tiny. We used the same hardware equipment for comparison experiments under the semi-self-constructed experimental protective equipment dataset. The comparison results are presented in Table 2.

**Table 2:** Comparison with current mainstream detection algorithms.

Detection Algorithms	P (%)	R (%)	Micro-F1 (%)	mAP (%)	FPS (f/s)
RetinaNet	53.6	36.9	43.7	33.4	6.8
Faster-RCNN	65.8	71	68	57.4	5
FCOS	74.6	80.8	77.6	69.7	7.2
YOLOv5	88.6	67	76	64.5	7.6
YOLOv7-tiny	85.1	75	80	71.7	9.7
YOLOv7	91.2	84	87	81.9	10.9
Ours	89	89	89	84.2	10.1

From the above table, it can be seen that after improvement the algorithm Micro-F1 score is 89 % and mAP is 84.2 %. Compared with the current mainstream detection algorithms, respectively, 2.3 %, 12.5 %, 19.7 %, etc. higher. A comprehensive comparison reveals that our algorithm is significantly superior to other existing detection algorithms in detecting experiment protective equipment while considering detection speed.

### 5.5 Ablation experiments

In order to verify the effectiveness of the improvement method, this paper designs four different models for experimental analysis, containing two ablation factors, namely the GAM global attention mechanism and the NWD small target metric detection algorithm. There are four different optimization and improvement models: (1) the original YOLOv7 network model; (2) the network model that introduces the GAM attention mechanism; (3) the network model that introduces the NWD small target metric; and (4) the network model designed in this paper.

The experimental results are shown in Table 3.

(1) Adding the GAM attention mechanism only in the YOLOv7 backbone network improves mAP by 1.4 % and Micro-F1 score by 1 %. It can be seen that adding the GAM global attention mechanism is able to combine global and local features although the model training steps are increased. The model can better capture the feature information of the extracted multi-category targets, which improves the detection accuracy and stability of the model.
(2) When the lightweight NWD small target loss function metric is introduced into the YOLOv7 model only, mAP and FPS are improved. This fully demonstrates the effectiveness of the NWD loss function small targets in detecting protective equipment, which makes the model more lightweight, enabling the test accuracy and inference speed to be improved.
(3) Introducing GAM and NWD into the YOLOv7 network structure, mAP is improved by 2.3 %, P and R reach equilibrium, and Micro-F1 score is improved by 2 %. It can be seen that the reliability and stability of the improved YOLOv7 model are enhanced while guaranteeing the detection speed, which makes the detection of PPE with higher accuracy realized.

**Table 3:** Comparison of the results of the ablation experiments for the improved section.

Detection Algorithms	GAM	NWD	P (%)	R (%)	Micro-F1 (%)	mAP (%)	FPS (f/s)
YOLOv7			**91.2**	84	87	81.9	10.9
YOLOv7	✓		90.2	86	88	83.3	9.9
YOLOv7		✓	89.3	85	87	83.1	**11.2**
YOLOv7	✓	✓	89	**89**	**89**	84.2	10.1

## 6 Discussion

### 6.1 Interpretation of the findings

This study analyses the application of target detection techniques for laboratory protective equipment identification, and finds that the YOLOv7-based target detection algorithm is more accurate in small target detection, with a range of mAP values all above 80 %. The experimental results show that, compared with the YOLOv7 algorithm, when only the GAM global attention mechanism is added to it, the mAP is improved by 1.4 %, and the Micro-F1 score is improved by 1 %; when only the lightweight NWD small-target loss function metrics are introduced to it, the mAP FPS is improved; when GAM and NWD are added to the YOLOv7 backbone, the mAP is improved by 2.3 %, P and R reach equal equilibrium, and the Micro-F1 score is improved by 2 %. The results further demonstrate that the addition of GAM and NWD modules to the YOLOv7 model solves the problem of difficult detection of small targets of protective equipment in complex scenarios in the laboratory and improves the overall performance.

### 6.2 Implications

Firstly, the experiments and methods proposed in this study construct a laboratory protective equipment data set containing a large number of small targets. This provides rich and high-quality data to support the in-depth research of existing methods, and promotes the applied research on the detection of small targets of protective equipment in different fields and from different perspectives. Second, this paper proposes a laboratory protective equipment identification method based on the improved YOLOv7 algorithm. By introducing GAM into the ELAN structure of the backbone network, a kind of ELAN-G module is constructed that considers both global and local features, which enhances the feature extraction capability of the network for small targets of laboratory protective equipment. The NWD small target loss function metric is used to replace the CIoU loss function of YOLOv7, which reduces the training loss of the model while improving the detection accuracy of the model, thus reducing the occurrence of missed detection. This method solves the problem of difficult detection of small targets of protective equipment in complex scenarios in laboratories, and provides a more intelligent and innovative solution to improve the level of laboratory safety management. This study helps to further improve the laboratory safety management system.

### 6.3 Strength and limitations

The algorithm proposed in this study performs well in terms of model loss, test accuracy and wearability specification detection on self-built datasets. The algorithm in this paper is advanced and superior for laboratory PPE detection. The algorithm in this paper achieves good results on the Laboratory Protective Equipment dataset, but further improvements are needed in terms of the diversity of dataset categories and the lightness of the algorithm. And the dataset is relatively homogeneous in terms of application scenarios, and can also be extended with actual scenario data, PPE types, and wearing scenarios.

### 6.4 Future work

In future work, the lightweight structure will be further investigated. The network will be optimized by techniques such as network pruning and distillation to improve its inference speed. The model will be able to achieve high efficiency recognition in the detection of other PPE as well, further promoting the research and application in improving the laboratory safety management system. The model will be deployed to the hardware equipment, which will eventually further achieve the effect of double guarantee of network model and detection accuracy.

The proposed algorithm is useful for automatically detecting of PPE in laboratories. Additionally, improved applications for identifying and prediction of personnel in hospitals, supermarkets, and other public places are expected and will be the focus of our future research.

## 7 Conclusion

This study is based on the laboratory safety management of some of the current reality: due to omission, less wear, irregular wear PPE behavior to the laboratory operator's personal safety brings great potential danger, and the current laboratory PPE normative wear detection work is mainly by the laboratory safety administrator visual supervision to complete. Based on this, combined with video monitoring, the computer is used to detect and identify the irregularities of PPE wearing, so as to liberate manpower and improve the efficiency of laboratory safety management. In this paper, we propose a small target detection and identification method for laboratory PPE based on the improved YOLOv7, which introduces GAM to the ELAN module of Backbone in YOLOv7, constituting the ELAN-G module, so that the model takes into account both global and local features. NWD optimization is used to replace the CIoU loss function of YOLOv7 to ensure the performance of the model while improving the execution efficiency of the model. The final results show that the improved model improves the checking rate by 5 %, the Micro-F1 score by 2 %, and the mAP by 2.3 % compared to the original model.

Although the improved YOLOv7 algorithm proposed in this paper has achieved better detection results, new research results are constantly emerging in the field of deep learning. In the future, more advanced network structures and training techniques can be explored, and further research will be conducted on lightweight structures to optimize the network through network pruning, distillation, and other techniques to improve its inference speed, so that the model can also achieve high efficiency recognition in the detection of other PPE to improve detection speed and accuracy.

**Ethics statement:** Due to the characteristics of the study, it does not require approval by the ethics committee. All participants provided informed consent for the publication of their images.

## Funding

This research was supported by the Innovation Group Project of 10.13039/501100019651Southern Marine Science and Engineering Guangdong Laboratory (Zhuhai) of China (No. 311021013).

## Data availability statement

The data that support the findings of this study are available from the corresponding author upon reasonable request.

## Consent for publication

All authors have given consent for publication.

## CRediT authorship contribution statement

**Huijuan Luo:** Writing – original draft, Validation, Software, Methodology, Investigation. **Wenjing Liu:** Writing – review & editing, Conceptualization. **Pinghu Xu:** Writing – review & editing. **Lijun Zhang:** Supervision, Resources, Funding acquisition, Conceptualization. **Lin Li:** Writing – review & editing, Project administration.

## Declaration of competing interest

The authors declare that they have no known competing financial interests or personal relationships that could have appeared to influence the work reported in this paper.
